# The Regulation of Skeletal Muscle Protein Turnover during the Progression of Cancer Cachexia in the *Apc^Min/+^* Mouse

**DOI:** 10.1371/journal.pone.0024650

**Published:** 2011-09-19

**Authors:** James P. White, John W. Baynes, Stephen L. Welle, Matthew C. Kostek, Lydia E. Matesic, Shuichi Sato, James A. Carson

**Affiliations:** 1 Department of Exercise Science, University of South Carolina, Columbia, South Carolina, United States of America; 2 Department of Medicine, University of Rochester Medical School, Rochester, New York, United States of America; 3 Department of Biological Sciences, University of South Carolina, Columbia, South Carolina, United States of America; Johns Hopkins University School of Medicine, United States of America

## Abstract

Muscle wasting that occurs with cancer cachexia is caused by an imbalance in the rates of muscle protein synthesis and degradation. The *Apc^Min/+^* mouse is a model of colorectal cancer that develops cachexia that is dependent on circulating IL-6. However, the IL-6 regulation of muscle protein turnover during the initiation and progression of cachexia in the *Apc^Min/+^* mouse is not known. Cachexia progression was studied in *Apc^Min/+^* mice that were either weight stable (WS) or had initial (≤5%), intermediate (6–19%), or extreme (≥20%) body weight loss. The initiation of cachexia reduced %MPS 19% and a further ∼50% with additional weight loss. Muscle IGF-1 mRNA expression and mTOR targets were suppressed with the progression of body weight loss, while muscle AMPK phosphorylation (Thr 172), AMPK activity, and raptor phosphorylation (Ser 792) were not increased with the initiation of weight loss, but were induced as cachexia progressed. ATP dependent protein degradation increased during the initiation and progression of cachexia. However, ATP independent protein degradation was not increased until cachexia had progressed beyond the initial phase. IL-6 receptor antibody administration prevented body weight loss and suppressed muscle protein degradation, without any effect on muscle %MPS or IGF-1 associated signaling. In summary, the %MPS reduction during the initiation of cachexia is associated with IGF-1/mTOR signaling repression, while muscle AMPK activation and activation of ATP independent protein degradation occur later in the progression of cachexia. IL-6 receptor antibody treatment blocked cachexia progression through the suppression of muscle protein degradation, while not rescuing the suppression of muscle protein synthesis. Attenuation of IL-6 signaling was effective in blocking the progression of cachexia, but not sufficient to reverse the process.

## Introduction

Skeletal muscle mass loss is a hallmark of cachexia, and muscle mass preservation is critical for the survival of many cancer patients [1]. Often cancer patients are not diagnosed until significant body weight loss has occurred [2], which limits treatment options in cachectic patients [3]. In contrast, patients diagnosed during initial stages of cachexia (<5% body weight loss) have a much better survival time and chemotherapy treatment outcomes [5]–[6]. Understanding the regulation of muscle wasting throughout the progression of cachexia is critical for developing both prevention and intervention strategies for treatment of cachexia [4]. Unfortunately, we have a limited understanding of muscle protein turnover regulation during the initial stages of cachexia. Furthermore, the progression of muscle wasting accelerates during the progression of cachexia [5]. This non-linear process creates gaps in our knowledge, which is based largely on regulatory changes during the later stages of cachexia.

The cellular signaling that disrupts the delicate balance between the rates of muscle protein synthesis and degradation is thought to be a vital foundation needed for a better mechanistic understanding of muscle wasting with cancer. While accelerated muscle protein degradation has acknowledged importance for the progression of wasting, the regulation of proteolytic mechanisms throughout the progression of cachexia remains uncertain. Muscle proteolysis, primarily through ubiquitin dependent mechanisms, is increased during late stage cachexia [6], while a single report has reported no difference in muscle proteolysis during the initial stages of cachexia in tumor bearing mice [7]. Similarly, the role of protein synthesis during the progression of cachexia is uncertain. While reduction in protein synthesis has been shown in patients with late stage cachexia [8], and in tumor bearing mice having at least a 16% reduction in body weight [7], the regulation during the initial stages of cachexia and eventual transition to severe weight loss warrants further exploration.

The muscle's capacity to synthesize protein is responsive to many stimuli including energy status, anabolic hormones, catabolic hormones, and loading [9]. The insulin-like growth factor-1 (IGF-1) signaling through PI3K/Akt/mTOR pathway can integrate feedback from a variety of growth-related stimuli to regulate myofiber size [10]. Circulating IGF-1 and muscle IGF-1 gene expression are generally decreased with wasting conditions [11] including cachexia [12], [13]. In addition, muscle mTOR activation is also reduced after at least a 12% loss of body weight and decreases further during late stage cachexia [14], [15]. IGF-1 signaling can also regulate muscle protein degradation through the suppression of forkhead box O (FOXO), whose transcriptional targets include muscle atrogin-1/MAFbx, Muscle RING Finger-1 (MuRF1) and autophagy-related genes [16], [17], [18]. Muscle 5′-adenosine monophosphate-activated protein kinase (AMPK), a sensor of cellular energy status, also regulates protein synthesis [19], [20], [21]. AMPK is activated by low cellular energy status and also muscle contraction [22]. AMPK can inhibit mTOR signaling through several mechanisms, including phosphorylation of raptor at Ser792 [23], which prevent binding and subsequent phosphorylation of p70S6K and 4EBP1. In addition, AMPK activation has shown to increase protein degradation in myotubes, being associated with an increase in FOXO transcriptional targets atrogin1 and MuRF1 [24]. In tumor bearing rats and mice, the activation of AMPK during the progression of cachexia has shown to be variable and its role in the regulation of muscle wasting with cachexia is difficult to interpret [25]. The present study seeks to clarify the role of AMPK in the regulation of muscle protein turnover during cachexia.

There is emerging evidence for lysosomal/autophagy to play a role during muscle wasting disease [26], [27]. Similar to other methods of muscle degradation, autophagy is an essential process in skeletal muscle required to remove organelles, i.e. mitochondria and portions of the cytoplasm [28]. Deletion of Atg7, a critical gene involved in autophagy, results in skeletal muscle atrophy, abnormal mitochondria and disorganization of sarcomeres [29]. In contrast to the ubiquitin system, autophagy is non-specific, ATP-independent process, which digests portions of the cell rather than individual proteins. The molecular components of autophagy/lysosomal pathways are well described, however, the regulation is not well known. Several genes have been identified as autophagy-related including, LC3β, Gabarpl1, Atg12l, PI3kIII, Ulk2, Atg4β and Beclin1. There have been reports that show autophagy-related genes increase during muscle dystrophy [30], diabetes [31], sepsis-induced wasting [27] and cancer related cachexia [31], [32].

Pro-inflammatory cytokine IL-6 has been implicated in the regulation of muscle wasting during cachexia in both humans and rodents [33]. Transgenic mice over-expressing IL-6 have muscle atrophy associated with increased expression of lysosomal and ubiquitin-related mRNA and proteins [34], [35]. In humans, IL-6 administration can cause a reduction in skeletal muscle protein synthesis [36]. Because of the catabolic effects that may be mediated through IL-6 during cachexia, several therapies have been proposed to inhibit IL-6 activity to prevent the progression of cachexia. The administration of an IL-6 receptor antibody has effectively countered muscle wasting in tumor bearing mice [36], [37]. Furthermore, the inhibition of IL-6 activity can reduce both ubiquitin and lysosomal degradation pathways in the gastrocnemius muscle of C-26 tumor bearing mice [37]. These data suggest that IL-6 inhibition can reduce muscle protein degradation; however, the effect on muscle protein synthesis has not been explored. Further work is needed to elucidate the associated between IL-6 signaling and muscle protein synthesis during the progression of cachexia. We have previously reported muscle wasting in the *Apc^Min/+^* mouse to be dependent on circulating pro-inflammatory cytokine IL-6 [38], [39]. However, the regulation of protein turnover during the initiation and progression to more severe loss has not been well studied during cachexia. The purpose of this study was to determine IL-6 regulation of muscle protein turnover during the initiation of and progression towards more severe cachexia in the *Apc^Min/+^* mouse. We directly measured muscle protein synthesis, muscle protein degradation, and associated signaling during different stages of weight loss. We also examined if IL-6 receptor signaling was important for the regulation of these processes during the progression of cachexia. Related to processes initiating cachexia, we hypothesized that protein synthesis would be reduced only during late stages of cachexia, while ATP dependent protein degradation would be primarily responsible for the initiation of muscle loss. After the initiation of cachexia, we hypothesized that the suppression of IL-6 signaling would rescue muscle mass through the induction of muscle protein synthesis and repression of ATP dependent protein degradation.

## Methods

### Animals

The University of South Carolina's Institutional Animal Care and Use Committee approved all animal experimentation in this study. *Apc^Min/+^* mice on a C57Bl/6 background were originally purchased from Jackson Laboratories (Bar Harbor, ME) and bred at the University of South Carolina's Animal Resource Facility as previously described [40]. Male *Apc^Min/+^* (n = 21) mice between 14 and 20 weeks of age were group housed and sacrificed at ages that provided stratification of body weight loss to allow the study of the progression of cachexia. The 4 groups used in this study were weight stable (WS; n = 5), initial (≤5%; n = 6), intermediate (6–19%; n = 4), and extreme (≥20%; n = 6) degrees of body weight loss, when compared to peak body weight. To block the progression of cachexia, a separate set of *Apc^Min/+^* mice were treated with an IL-6 receptor antibody (n = 5) or PBS control (n = 7) for two weeks, starting at after the onset of cachexia (16 weeks). Wild-type C57Bl/6 controls were also treated with the IL-6 receptor antibody (n = 6) or PBS control (n = 6) at 16 weeks. The room was maintained on a 12∶12 light∶dark cycle with the light period starting at 0700. Mice were provided standard rodent chow (Harlan Teklad Rodent Diet, #8604, Madison, WI) and water *ad libitum*.

### Muscle Collection

Mice were given a subcutaneous injection of ketamine/xylazine/acepromazine cocktail (1.4 ml/kg BW) before the gastrocnemius was dissected. Tibia length was measured as an indicator of animal body size and a correction factor for skeletal muscle weights. Thirty minutes prior to sacrifice, all mice were given an intraperitoneal injection of 150 mM ^2^H_5_-phenylalanine (Cambridge Isotope Laboratories) in a 75 mM NaCl solution at a dose of 2 ml/100 g body weight [41]. At sacrifice, the gastrocnemius muscles were rinsed in PBS, snap frozen in liquid nitrogen, weighed, and stored at −80°C until further analysis.

### Intestinal Tissue Collection

Intestinal tissue collection was performed as described previously with a slight modification [39], [42], [43], [44]. Briefly, the small intestines were carefully cut at the distal end of the stomach and at the proximal end of the cecum. The large intestine was removed from the distal end of the cecum to the anus. Mesentery adipose tissue was removed with forceps and the small intestine was cut into four equal sections. All intestinal sections were flushed with PBS, opened longitudinally with a pair of scissors, and flattened with a cotton swab between two pieces of blotting paper. Intestinal sections were fixed in 4% paraformaldehyde (PFA) in PBS overnight and transferred to PBS for storage at 4°C for further analysis.

### Polyp Counts

Polyp counts were performed as previously described [39], [42], [43], [44]. Briefly, 4% PFA-fixed intestinal sections from all animals were briefly stained in 0.1% methylene blue, and they were placed under a dissecting microscope. Polyps were counted by the same investigator in a blind manner. Polyps were categorized as large (>2 mm in diameter), intermediate (1∼2 mm) or small (<1 mm).

### IL-6 Receptor Antibody Administration

The MR16-1 IL-6 receptor antibody was a generous gift from Chugai Pharmaceutical CO., LTD, Tokyo, Japan. The antibody was administered at a dose of 300 ug/mouse in phosphate buffered saline by intraperitoneal injection every three days for two weeks starting at 16 weeks of age. PBS was injected as a control vehicle.

### Myofibrillar Protein Synthesis

Gastrocnemius muscle samples were homogenized in 1 ml water. Myofibrils and other insoluble proteins were pelleted by centrifugation, and the supernatants containing free amino acids were used to determine the ratio of free ^2^H_5_-phenylalanine (m/z 239 fragment) to endogenous (unlabeled) phenylalanine (m/z 234 fragment). The ratios were determined by GC-mass spectrometric analysis of the *t*-butyldimethylsilyl derivatives of these amino acids. Myofibrillar proteins were washed, hydrolyzed, and analyzed for ^2^H_5_-phenylalanine enrichment by monitoring the m/z 237 and 239 fragments, as described in detail by Welle et al. [41].

The fractional rate of myofibrillar synthesis, % per day, was calculated as the % enrichment of tracer in the hydrolysate of myofibrillar protein, divided by the tracer enrichment in the free amino acid pool of muscle tissue. Myofibrillar protein enrichment was determined from the m/z 237 and m/z 239 ions because the lightest isotopomer (m/z 234) saturated the MS detector. The myofibrillar/free enrichment ratio was multiplied by 48 to obtain %/day values because tracer incorporation occurred over a period of 30 min.

### Protein Degradation

To assay the degradation of soluble muscle protein, a modified proteasomal proteolysis assay reported by Hu et al [45] was be used. The gastrocnemius muscle was removed and frozen in liquid nitrogen. Muscle extracts (∼40 mgs) were homogenized in ice-cold harvest buffer (5 mm Tris-HCl (pH 8.8), 1% glycerol, 1 mm EDTA, 1 mm EGTA, freshly constituted 1 mm β-Me, and 50 mm EP-475). The homogenates were centrifuged at 30,000× *g* for 30 min, and then the soluble fractions were used to measure protein degradation. For the protein degradation determination, muscle extracts were dialyzed against a basal buffer [20 mmTris-HCl (pH 7.6) 10% glycerol, 2 mm dithiothreitol (DTT), 10 mm magnesium acetate, and 20 mm potassium chloride] to remove accumulated tyrosine. Aliquots of extracts were incubated for 2 h at 37 C with or without an ATP-generation system (1 mm ATP, 100 µg/ml creatine kinase, and 10 mm phosphocreatine); ubiquitin (250 µg/ml) was also added. The reaction was stopped with trichloroacetic acid, precipitated proteins were removed by centrifugation, and free tyrosine was measured fluorometrically (450 nm excitation/550 nm emission) to calculate the rate of protein degradation [46].

### AMPK Activity

AMPK activity was determined using a kit from Linco (St. Louis, MO). In brief, whole muscle homogenates were added to a 96-well plate coated with AMPK substrate IRS-1, incubated for 2 hr, followed by addition of a secondary antibody, conjugated to horseradish peroxidase (HRP) and specific for phosphorylated Ser 794 in IRS-1. Lastly, illuminating agent was added to the plate to quantify phosphor-IRS-1 and determine AMPK activity.

### RNA Isolation, cDNA Synthesis, and Real Time PCR

RNA isolation, cDNA synthesis, and real-time PCR were performed as previously described [47], using reagents from Applied Biosystems (Foster City, CA). Fluorescence labeled probes for IGF-1, skeletal alpha actin, C2 proteasomal subunt, C7 proteasomal subunit, atrogin1, murf1 (FAM dye) and the ribosomal RNA 18s (VIC dye) were purchased from Applied Biosystems and quantified with TaqMan Universal mastermix. Data were analyzed by ABI software using the cycle threshold (C_T_), which is the cycle number at which the fluorescence emission is midway between detection and saturation of the reaction.

### Western Blotting

Western blot analysis was performed as previously described [48]. Briefly, frozen gastrocnemius muscle was homogenized in Mueller buffer and protein concentration determined by the Bradford method [49]. Crude muscle homogenate 40 µg was fractionated on 6%–15% SDS-polyacrylamide gels. Gels were transferred to PVDF membranes overnight. Membranes were stained with Ponceau red to verify equal loading of each gel. Membranes were blocked overnight in 5% non-fat milk in Tris-buffered saline with 0.1% Tween-20 (TBS-T). Primary antibodies for p-Akt (Ser473), Akt, p-4EBP1 (Ser65), 4EBP1, p-mTOR (Ser2448), mTOR, p-S6K (Thr389), S6K, p-AMPK (Thr172), AMPK, p-Stat3 (Tyr705), Stat3, p-Raptor (Ser792), Raptor, Beclin-1, Atg7, LC3β, Ubiquitin, atrogin1 (Cell signaling) and SOCS3 (Santa Cruz) were diluted 1∶1000 to 1∶500 in 5% milk in TBS-T followed by 1 hour incubation with membranes at room temperature. Anti-rabbit IgG horseradish-peroxidase conjugated secondary antibody (Cell Signaling) was incubated with the membranes at 1∶2000 dilutions for 1 hour in 5% milk in TBS-T. Enhanced chemiluminescence (ECL) (GE Healthcare Life Sciences, Piscataway, NJ) was used to visualize the antibody-antigen interactions. Images were digitally scanned and blots were quantified by densitometry using scientific imaging software (Scion Image, Frederick, MD).

### Statistical Analysis

A one way ANOVA was used to determine differences in *Apc^Min/+^* mice with varying degrees of body weigh loss. A two way ANOVA was used to determine differences between genotype and IL-6 receptor antibody treatment. Post-hoc analyses were performed with Student-Newman-Keuls methods. A pre-planned t test was used to compare wild-type mice with weight stable *Apc^Min/+^* in Table 1. Significance was set at p<0.05.

**Table 1:** Body weight, tissue mass and plasma IL-6 concentrations in wild-type and *Apc^Min/+^* mice grouped by percentage of body weight loss.

		Peak	Sac						
		BW	BW	% Change	Gastroc	Epi Fat	Tibia Length	IL-6	Spleen
Group		(g)	(g)		(mg)	(mg)	(mm)	(pg/ml)	(mg)
Wt	n = 5	25.4±0.3	25.3±0.4	0.1±0.4	121.4±1.8	391±27	16.9±0.1	0±0	102±11
*Apc^Min/+^*									
WS	n = 5	25.4±0.8	25.6±0.9	0.0±0.0	121.5±5.6	330±23	17.0±0.1	2.8±2	250±42*
≤5%	n = 6	23.4±0.2	22.5±0.2†	−4.1±0.6†	100.5±2.8†	122±26†	16.7±0.1	30±3†	425±13†
6–19%	n = 4	24.8±0.6	21.3±0.8†	−13.9±1.5†,&	82.3±3.8†,&	6.7±6.7†,&	17.1±0.1	108±36†,&	480±92†
≥20%	n = 6	25.2±0.5	19.8±0.3†	−21.4±0.6$	68.6±3.1$	0±0$	17.3±0.1	92±23†,&	379±41†

Wild-type mice at 16 weeks of age were used as non tumor bearing control. Values are means ± SE.

† Signifies different from WS.

& Signifies different from WS and <5% groups.

$ Signifies difference from all groups.

*Signifies different from wild-type mice.

WS, weight stable. Epi, Epididymal fat pad. Sac, sacrifice, BW, body weight, Wt, wild-type mice.

## Results

### Body weight, fat mass and inflammatory state during the progression of cachexia in the *Apc^Min/+^* mouse

Mice were sacrificed between 14 to 20 weeks of age, which is the age range for cachexia development in *Apc^Min/+^* mice, and categorized according to percent body weight loss at the time of sacrifice compared to their peak body weight. The groups were designated as weight stable (no weight loss), ≤5% body weight loss (initial), 6–19% weight loss (intermediate) and ≥20% loss (extreme). Weight stable *Apc^Min/+^* mice, (14 weeks of age), had body weights similar to aged-matched wild-type mice (Table 1). During the initiation of cachexia, circulating IL-6 (*P* = 0.02; Table 1) and spleen weight (*P*<0.001) were increased slightly, compared to weight stable *Apc^Min/+^* mice, and increased further with the progression to intermediate body weight loss (*P*<0.001; Table 1). There was no further change in circulating IL-6 or spleen weight during the progression from intermediate to extreme body weight loss. Epididymal fat mass was reduced by 63% (*P*<0.001; Table 1) during the initiation of cachexia and continued to decrease with the progression of cachexia (*P*<0.001; Table 1). Tibia length, an index of overall body size, was not different between any groups (Table 1) indicating that normal bone growth was occurring during the progression of cachexia.

### Myofibrillar protein synthesis is reduced during the initiation of cachexia

**Figure 1:**
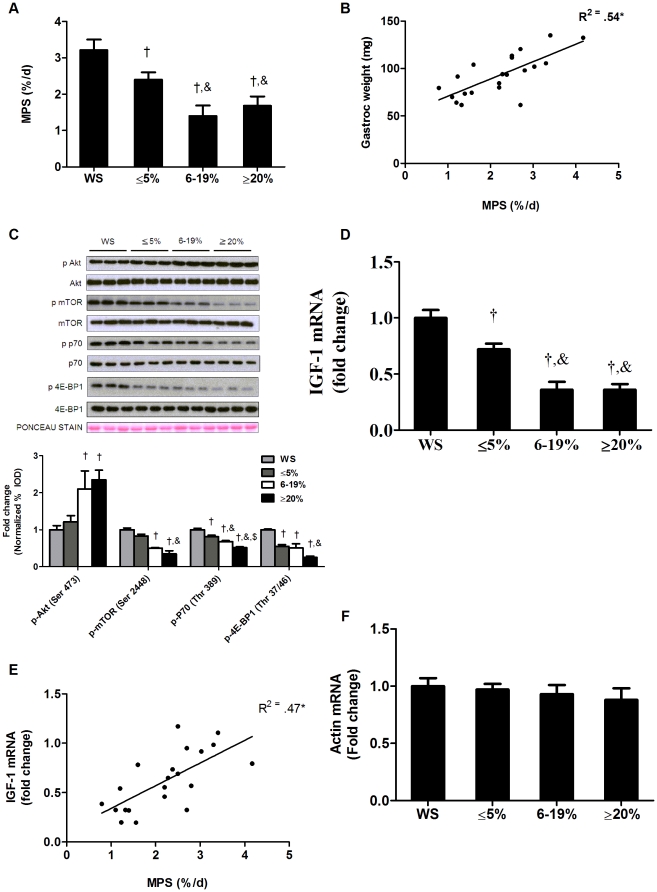
Muscle protein synthesis and IGF-1/mTOR signaling are reduced during the progression of cachexia in *Apc^Min/+^* mice. Protein synthesis and IGF-1 expression were measured in *Apc^Min/+^* mice during the progression of cachexia. A) Myofibrillar protein synthesis. B) Correlation between muscle weights and protein synthesis. C) *Upper*: representative western blot of phosphorylated and total forms of Akt (Ser 473), mTOR (Ser2448), p70S6k (Thr389) and 4EBP-1 (Thr37/46). *Lower*: The ratio of phosphorylated and total Akt, mTOR, p70 and 4EBP1 in the gastrocnemius muscle normalized to the WS group. D) IGF-1 expression and E) correlation between IGF-1 gene expression and protein synthesis. F) Skeletal alpha actin mRNA expression. Values are means ± SE. Significance was set at p<0.05. † Signifies different from WS mice. & Signifies difference from mice with ≤5% body weight loss. $ Signifies difference from mice with 6–19% body weight loss. WS, weight stable.

During the initiation of cachexia, *Apc^Min/+^* mouse gastrocnemius muscle mass was decreased 17% (*P* = 0.001; Table 1), and as cachexia progressed muscle mass continued to decline (*P*<0.001; Figure 1A). During the initiation of cachexia there was a corresponding 19% reduction in the rate of myofibrillar protein synthesis (*P* = 0.001; Figure 1A), which continued to decrease further with intermediate weight loss (*P*<0.001). Unlike muscle mass loss, there was no further decrease in the rate of myofibrillar protein synthesis between *Apc^Min/+^* mice exhibiting intermediate or extreme weight loss. Gastrocnemius muscle mass was correlated (*P* = 0.003; Figure 1B) with myofibrillar protein synthesis rate in all groups of *Apc^Min/+^* mice. Muscle mass and protein synthesis prior to cachexia development were similar between wild-type and weight stable *Apc^Min/+^* mice (Figure S1A and B).

To evaluate changes in cellular signaling involved in the regulation of protein synthesis, we measured IGF-1/Akt/mTOR pathway activation during the initiation and progression of cachexia. Akt phosphorylation (Ser 473), typically associated with promoting muscle anabolism, was unchanged during initiation of cachexia. With the transition to intermediate and extreme weight loss we found muscle Akt phosphorylation to be increased approximately 2-fold (*P*<0.001; Figure 1C). During the initiation of cachexia, there was a trend for mTOR phosphorylation on Ser residue 2448 to decrease (*P* = 0.06; Figure 1C), while phosphorylation of mTOR targets, p70 (Thr389) and 4EBP1 (Ser65), were reduced 20% (*P* = 0.001; Figure 1C) and 55% (*P*<0.001) during initial stages of cachexia, respectively. With the transition to intermediate weight loss, the mTOR (−50%), and p70 (−37%) phosphorylation were further reduced (*P*<0.001; Figure 1C). With extreme body weight loss only p70 phosphorylation had a further decrease (Figure 1C).

Muscle IGF-1 mRNA expression decreased 28% (*P* = 0.001; Figure 1D) during the initiation of cachexia. Although IGF-1 mRNA expression was further decreased with intermediate weight loss (Figure 1D), there was no further reduction in muscle IGF-1 expression with extreme weight loss. Muscle IGF-1 expression and the rate of myofibrillar protein synthesis were correlated across all weight loss groups (*P* = 0.03; Figure 1E). Muscle IGF-1 expression in weight stable *Apc^Min/+^* and wild-type mice were similar (Figure S1C). Despite the reduction in myofibrillar protein synthesis, we found no effect of cachexia on skeletal alpha actin mRNA expression (Figure 1F) implying that the reduction in myofibrillar protein synthesis was not due to a reduction in mRNA availability.

### Muscle AMPK phosphorylation is induced during extreme body weight

**Figure 2:**
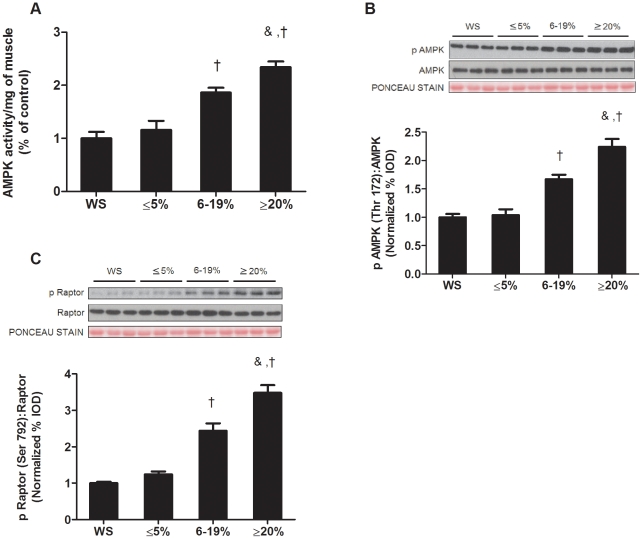
AMPK activation increases during late stage cachexia. AMPK activation was measured in *Apc^Min/+^* mice during the progression of cachexia. A) AMPK activity in the gastrocnemius muscle normalized to the WS mice. B) *Upper*: representative western blot of phosphorylated AMPK (Thr172) and total AMPK in the gastrocnemius. *Lower*: The ratio of phosphorylated to total forms of AMPK in the gastrocnemius muscle. C) *Upper*: representative western blot of phosphorylated raptor (Ser792) and total raptor in the gastrocnemius. *Lower*: The ratio of phosphorylated to total forms of raptor in the gastrocnemius muscle. Values are means ± SE. Significance was set at p<0.05. † Signifies different from WS mice. & Signifies difference from mice with ≤5% body weight loss. $ Signifies difference from mice with 6–19% body weight loss. WS, weight stable.

Muscle AMPK phosphorylation (Thr 172) and activity were not changed during the initiation of cachexia (Figures 2A and 2B). As cachexia progressed, both AMPK phosphorylation and activity were increased with intermediate weight loss, and further increased with extreme weight loss (Figures 2A and 2B). AMPK activity and phosphorylation status were similar between wild-type and *Apc^Min/+^* prior to the development of cachexia (Figure S2A and B). AMPK can inhibit mTOR activity through several mechanisms, one being phosphorylation of raptor at Ser792. Alterations in raptor phosphorylation coincided with AMPK phosphorylation during the development of cachexia. Raptor phosphorylation was not increased during the initiation of cachexia, but increased with intermediate weight loss and was further increased with extreme weight loss (Figure 2C).

### Muscle protein degradation is regulated by both ATP dependent and independent processes

**Figure 3:**
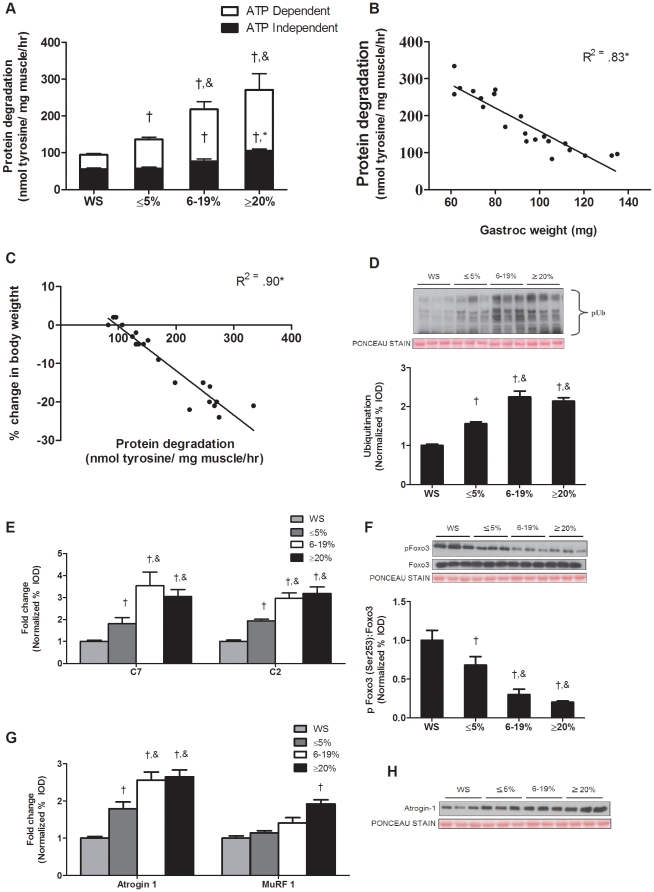
Skeletal muscle protein degradation consists of ATP dependent and independent mechanisms during the progression of cachexia in the *Apc^Min/^* mice. Protein degradation was measured in *Apc^Min/+^* mice with progressive body weight loss. A) Protein degradation determined by tyrosine release assay. White bars represent ATP dependent degradation; Black bars represent ATP independent degradation. B) Correlation between protein degradation and gastrocnemius weight. C) Correlation between protein degradation and the percentage of body weight loss. D) *Upper* representative western blot of ubiquitinated proteins in the gastrocnemius muscle. *Lower* Quantification of ubiquitinated proteins normalized to weight stable *Apc^Min/+^* mice. E) Gene expression of C7 and C2 proteasomal subunits. F). *Upper*: representative western blot of phosphorylated and total forms of Foxo3. *Lower*: The ratio of phosphorylated and total Foxo3. G) Gene expression of atrogin1 and MuRF1. H) Representative western blot of atrogin1 protein. Values are means ± SE. Significance was set at p<0.05. † Signifies different from WS groups. & Signifies difference from mice with ≤5% body weight loss. WS, weight stable.

Total protein degradation, determined by tyrosine release assay was not different between wild-type and weight stable *Apc^Min/+^* mice (Figure S3). However, total degradation was increased by 45% (*P* = 0.001; Figure 3A) in mice with initial weight loss while degradation was increased 134% and 188% during intermediate and extreme body weight loss respectively. The initial increase in protein degradation was due to ATP dependent degradation. During extreme cachexia, there was an increase in both ATP dependent and independent degradation. In contrast to ATP dependent degradation, ATP independent degradation continued to increase with more severe body weight loss (Figure 3A). Total protein degradation strongly correlated with gastrocnemius mass within all groups of weight loss (*P*<0.001; Figure 3B). In addition, protein degradation was significantly correlated to the percentage of body weight loss in the *Apc^Min/+^* mice (*P*<0.001; Figure 3C). To further explore the role of ATP independent and dependent proteolytic pathways, we measured components of the ubiquitin dependent proteasomal pathway. During the initiation of cachexia, muscle protein ubiquitination increased 56% (*P* = 0.001; Figure 3D) and further increased (*P*<0.001) with greater body weight loss. C7 and C2 proteasomal subunit expression increased by 94% (*P* = 0.001) and 81% (*P* = 0.008; Figure 3E) respectively during initiation of cachexia and further increased with the progression to extreme cachexia. There were no differences in ubiquitination, C7 or C2 expression between *Apc^Min/+^* mice with intermediate and extreme weight loss.

Akt signaling can also regulate muscle degradation pathway through phosphorylation and subsequent inhibition of the transcription factor Foxo3 at Ser 253. During the initiation of cachexia, Foxo3 phosphorylation was reduced by 39% (*P* = 0.001; Figure 3F), and further reduced during the transition to intermediate weight loss, but not further reduced with extreme body weight loss (Figure 3F). Atrogin-1 and MuRF1 mRNA expression, Foxo regulated muscle E3 ligases, demonstrated different expression patterns during the development of cachexia. Atrogin1 mRNA increased 79% (*P* = 0.01, Figure 3G) during the initiation of cachexia, and was further induced with intermediate and extreme weight loss. MuRF1 mRNA expression was not increased during initiation of cachexia or the transition to intermediate weight loss. However, *Apc^Min/+^* mice with extreme weight loss showed a 92% (*P* = 0.02, Figure 3G) increase in MuRF1 expression. Atrogin1 protein expression was increased by 79% (*P* = 0.004; Figure 3H) during initial body weight loss. Atrogin-1 protein expression was further induced with intermediate bodyweight loss, but there was no further induction with extreme loss of body mass.

**Figure 4:**
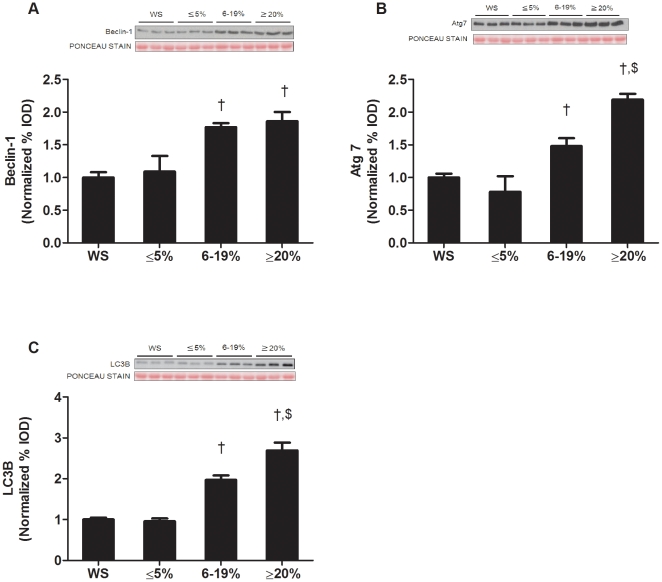
Autophagy is increased during late stage cachexia in the *Apc^Min/**+**^* mouse. A) *Upper* representative western blot of Beclin-1 protein. *Lower* Quantification of Beclin-1 protein normalized to weight stable *Apc^Min/+^* mice, B) *Upper* representative western blot of Atg7 protein. *Lower* Quantification of Atg7 protein. C) *Upper* representative western blot of LC3β protein. *Lower* Quantification of LC3β protein. Values are means ± SE. Significance was set at p<0.05. † Signifies difference from weight stable *Apc^Min/+^* mice. $ Signifies different from *Apc^Min/+^* mice with 6–19% body weight loss. WS, weight stable.

The role of autophagy during the development and progression of cachexia was explored by the examination of autophagy-related proteins. We did not observe any differences in autophagy-related protein expression between wild-type and weight stable *Apc^Min/+^* mice (Figure S4A and B). In support of our protein degradation measurements, which show non-ATP dependent protein degradation increased after the onset of cachexia, no autophagy-related proteins were induced during the initiation of cachexia. During the transition to intermediate body weight loss, the expression of muscle Beclin-1, Atg7, and LC3B proteins all increased (Figure 4A, B, & C). With extreme weight loss, muscle Atg 7 and LC3B protein expression had an even greater induction (Figure 4A, B, &C).

### IL-6 signaling inhibition after the initiation of cachexia suppresses the progression of cachexia by sparing muscle mass independent of changes in muscle protein synthesis

**Figure 5:**
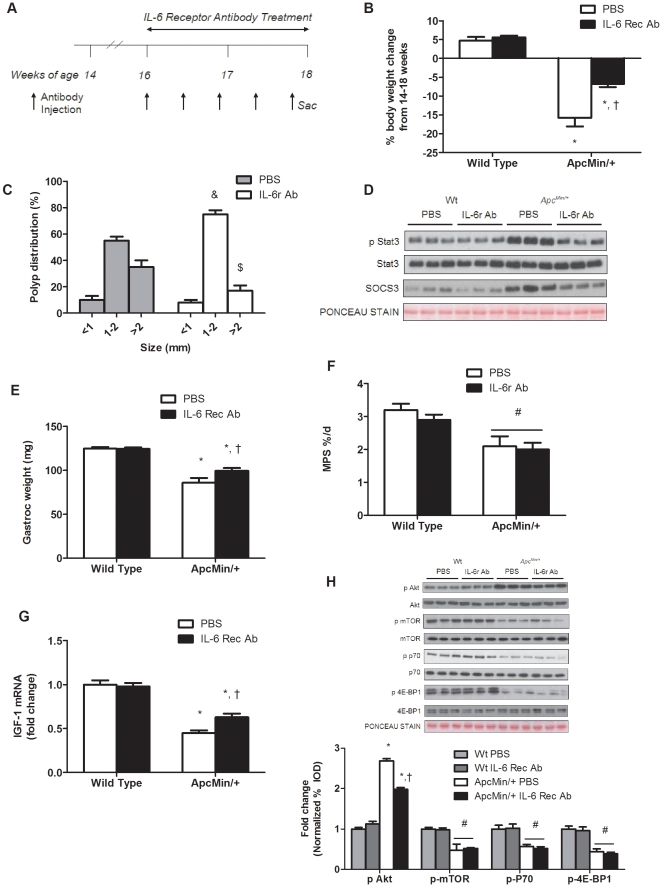
Inhibition of IL-6 signaling attenuates the progression of cachexia independent of changes in protein synthesis. Wild type and *Apc^Min/+^* mice were given an IL-6 receptor antibody or PBS control at 16 weeks of age and sacrificed at 18 weeks. A) Experimental design showing administration schedule and time line of IL-6 receptor antibody or PBS treatment. B) Percentage body weight change from peak body weight to sacrifice weight. C) Polyp distribution presented in percentage of total tumors. D) Representative western blot of phosphorylated and total forms of Stat3 and total SOC3 protein expression. E) Gastrocnemius weight and F) muscle protein synthesis measured at sacrifice. G) IGF-1 gene expression in the gastrocnemius muscle. H) *Upper*: Representative western blots of phosphorylated and total forms of Akt, mTOR, p70S6K, and 4E-BP1. *Lower*: The ratio of phosphorylated to total form of the given protein normalized to PBS treated wild-type mice. Values are means ± SE. Significance was set at p<0.05. *Signifies difference within treatment. † Signifies difference within genotype. # Signifies main effect of genotype. & Signifies difference in polyps 1–2 mm. $ Signifies difference between polyps >2 mm.

This experiment administered an IL-6 receptor (IL-6r) antibody to mice that had initiated cachexia and examined effects on the regulation of skeletal muscle protein turnover (See Research Design; Figure 5A). PBS treated *Apc^Min/+^* mice had a 2.8 (*P*<0.001, Figure 5D) and 2.4 fold (*P*<0.001) increase in muscle Stat3 activation and SOC3 protein expression. Treatment with the IL-6r antibody reduced muscle Stat3 activation by 50% and SOC3 expression by 41% (*P*<0.001; Figure 5D).

IL-6r antibody administration from 16 to 18 weeks of age attenuated the progression of cachexia. *Apc^Min/+^* mice receiving control PBS injections had 16% reduction in body weight (*P*<0.001; Figure 5B; Table 2) from their peak weight, while *Apc^Min/+^* mice receiving injections of IL-6r antibody only had a 7% loss in body weight (*P* = 0.004; Figure 5B; Table 2). In addition, gastrocnemius muscles from *Apc^Min/+^* mice receiving the IL-6r antibody were 16% (*P* = 0.01; Figure 5E) heavier than control *Apc^Min/+^* mice. IL-6r antibody administration had no effect on muscle or body weight of wild-type controls. The effects of the IL-6r antibody on the *Apc^Min/+^* mice were not due to changes in food consumption. Both wild-type and *Apc^Min/+^* mice consumed similar amounts of food throughout the study (Figure S5). However, the IL-6r antibody treatment did reduce the percentage of large polyps in *Apc^Min/+^* mice (*P* = 0.014; Figure 5C). Total polyp number was not affected by the IL-6r antibody treatment (Figure S6).

**Table 2:** The effect of an IL-6 receptor antibody on body weight and inflammatory markers in wild-type and *Apc^Min/+^* mice.

			Peak	16 week	18 week	14–16 week	14–18 week		
			BW	BW	BW	% change	% change	IL-6	Spleen
Genotype	Treatment		(g)	(g)	(g)			(pg/ml)	(mg)
Wt	PBS	n = 8	26.6±0.6	27.1±0.6	28.0±0.8	2.1±0.6	4.7±1.1	0±0	81.8±3
Wt	+IL-6r Ab	n = 8	26.5±0.5	27.3±0.5	28.1±0.6	2.8±0.3	5.6±0.5	0±0	82.3±3.4
*Apc^Min/+^*	PBS	n = 7	24.6±0.8#	23.5±0.8#	21.2±0.8#	−4.6±0.8#	−15.8±2.3*	64±6#	399±44#
*Apc^Min/+^*	+IL-6r Ab	n = 6	24.9±0.4#	24.0±0.5#	23.3±0.3# †	−3.6±0.7#	−6.8±0.8* †	55±9#	472±38#

Values are means ± SE.

# signifies main effect of genotype.

*signifies difference within PBS and IL-6RecAb groups.

† signifies difference from PBS treated ApcMin/+ group.

Wt, wild-type mice, IL-6r Ab, IL-6 Receptor Antibody, BW, body weight.

**Figure 6:**
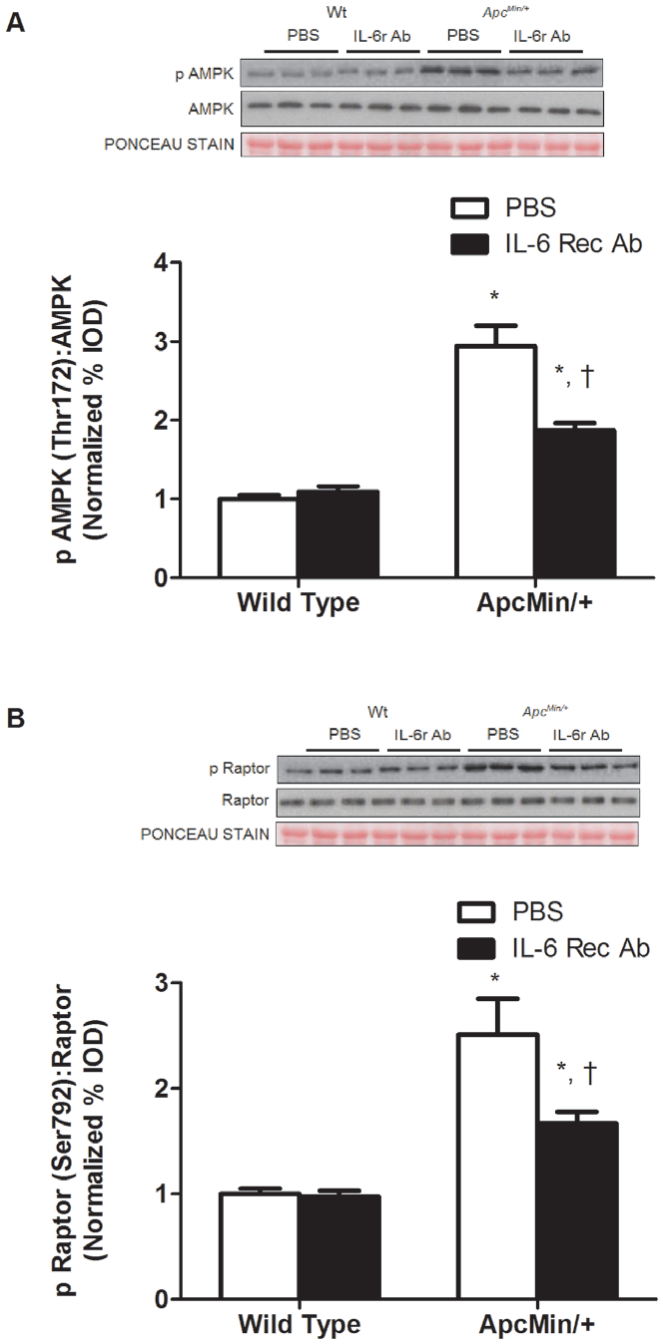
Administration of IL-6 receptor antibody attenuates the increase of phosphorylation of AMPK and raptor. A). *Upper*: representative western blot of phosphorylated and total forms of AMPK. *Lower*: The ratio of phosphorylated and total AMPK expression normalized to wild-type PBS control. B). *Upper*: representative western blot of phosphorylated and total forms of raptor. *Lower*: The ratio of phosphorylated and total forms of raptor. Values are means ± SE. Significance was set at p<0.05. *Signifies difference within treatment. † Signifies difference within genotype.

Related to protein synthesis, there was a main effect of genotype (*P* = 0.002; Figure 5F) for *Apc^Min/+^* mice to have a lower myofibrillar protein synthesis rate, compared to wild-type mice. Within *Apc^Min/+^* mice, the administration of the IL-6r antibody did not alter the attenuated rate of myofibrillar protein synthesis. Although IL-6r antibody treatment increased muscle IGF-1 mRNA expression 65% (*P*<0.001; Figure 5G), mTOR signaling was unaffected by IL-6r antibody treatment. As expected there were main effects of genotype (*P*<0.05; Figure 5H) for mTOR, p70S6K and 4E-BP1 phosphorylation, the *Apc^Min/+^* mice having suppressed signaling compared to wild-type mice. The cachexia induction of Akt phosphorylation was attenuated 30% (*P* = 0.001; Figure 5H) by the IL-6r antibody treatment. IL-6r antibody administration reduced AMPK and raptor phosphorylation (*P* = 0.001; Figure 6A, B) compared to PBS treated *Apc^Min/+^* mice. However, AMPK and raptor phosphorylation was still elevated above levels in non-cachectic muscle.

### IL-6 signaling inhibition after the initiation of cachexia can attenuate muscle protein degradation

**Figure 7:**
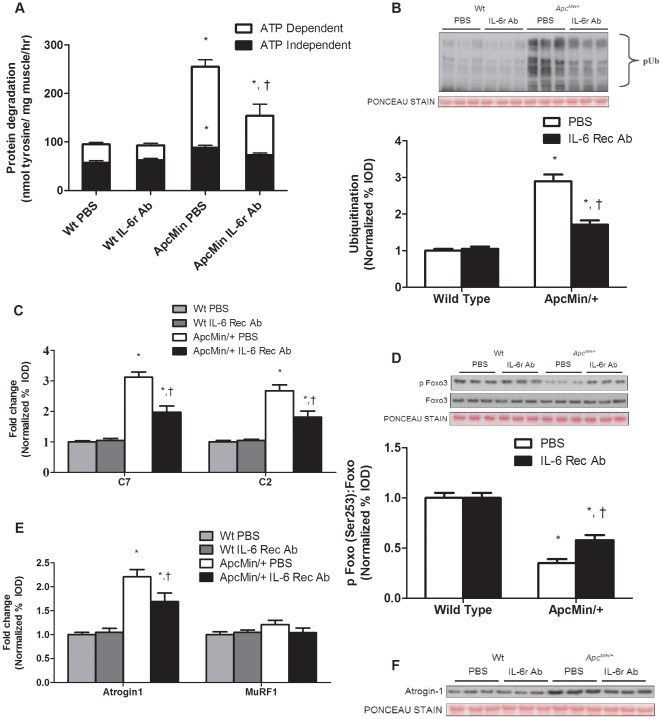
IL-6 inhibition by IL-6 receptor antibody attenuates protein degradation and ubiquitin proteasomal degradation. Wild type and *Apc^Min/+^* mice were given an IL-6 receptor antibody or PBS control at 16 weeks of age and sacrificed at 18 weeks. A) Protein degradation determined by tyrosine release assay. White bars represent ATP dependent degradation; Black bars represent ATP independent degradation. B) *Upper* representative western blot of ubiquitinated proteins in the gastrocnemius muscle. *Lower* Quantification of ubiquitinated proteins normalized to wild-type PBS control mice. C) Gene expression of C7 and C2 proteasomal subunits. D) *Upper*: representative western blot of phosphorylated and total forms of Foxo3. *Lower*: The ratio of phosphorylated and total Foxo3. E) Gene expression of atrogin1 and MuRF1. F) Representative western blot of atrogin1 protein. Values are means ± SE. Significance was set at p<0.05. *Signifies difference from wild-type control. † Signifies difference within *Apc^Min/+^* mice.

*Apc^Min/+^* control mice not treated with the IL-6r antibody increased total protein degradation by 2.5 fold (*P*<0.001; Figure 7A) during the progression of cachexia, which involved ATP dependent and independent mechanisms of degradation. Treatment with the IL-6r antibody attenuated total protein degradation in the *Apc^Min/+^* mouse by 40% (*P* = 0.002; Figure 7A). The primary reduction was in ATP dependent degradation, although ATP independent degradation was also attenuated. There was no effect of IL-6r antibody administration on protein degradation in wild-type mice. *Apc^Min/+^* mice that were not treated with the IL-6r antibody increased muscle protein ubiquitination approximately 3-fold (*P*<0.001; Figure 7B), and the antibody treatment significantly attenuated this induction. In addition, C7 and C2 proteasomal subunit expression was reduced by IL-6r antibody administration (Figure 7C). However, muscle protein ubiquitination and proteasomal subunit expression in *Apc^Min/+^* mice treated with the IL-6r antibody remained elevated when compared to non-cachectic muscle, which was expected since the treated mice had already initiated cachexia. These facts support the finding that the IL-6r antibody treatment was diminishing the rate of cachexia development, but was not reversing the process.

**Figure 8:**
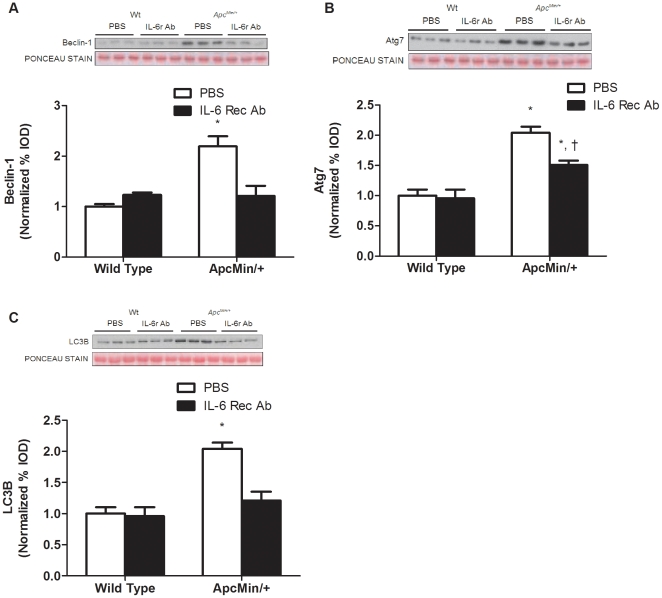
The induction of autophagy is attenuated by IL-6 inhibition. *A) Upper* representative western blot of Beclin-1 protein. *Lower* Quantification of Beclin-1 protein normalized to wild-type PBS control mice. B) *Upper* representative western blot of Atg7 protein. *Lower* Quantification of Atg7 protein normalized to wild-type PBS control mice. C) *Upper* representative western blot of LC3β protein. *Lower* Quantification of LC3β protein normalized to wild-type PBS control mice. Values are means ± SE. Significance was set at p<0.05. *Signifies difference from wild type control. † Signifies difference within *Apc^Min/+^* mice.

The IL-6r antibody treatment increased Foxo3 phosphorylation 66% (*P* = 0.004; Figure 7D), but this level was still attenuated compared to Foxo3 phosphorylation in non-cachectic mice. Although atrogin1 gene and protein expression were both attenuated (Figure 7E, F) by IL-6r antibody treatment, atrogin-1 gene and protein expression were still elevated when compared to non-cachectic muscle. MuRF1 expression was not different among groups (Figure 7E). The expression of autophagy-related proteins Beclin-1, Atg7 and LC3B increased approximately 2-fold during the progression of cachexia in untreated mice (*P*<0.001; Figure 8A,B,C). IL-6 receptor antibody treatment prevented the induction of Beclin-1 and LC3B expression (*P*<0.001; Figure 8A,C) in the *Apc^Min/+^* mouse and attenuated the increase in Atg7 by 25% (*P* = 0.03; Figure 8B).

## Discussion

Although the regulation of skeletal muscle protein turnover during cancer cachexia has been extensively investigated, the potential for differential regulation of myofibrillar protein synthesis and degradation during the transition from the initiation of weight loss to more severe loss is just beginning to be understood. Determining processes that first initiate and then allow for the progressive loss of muscle mass loss are critical for the eventual treatment of cancer cachexia, since recovery or reversal of cachexia will likely be far more difficult in the severely cachectic state. Our current study provides further evidence that a surge in circulating IL-6 is critical for the progression of cachexia in the *Apc^Min/+^* mouse. We report a 3–4 fold induction of IL-6 levels in mice transitioning from the initiation of muscle mass loss to more severe loss, and that administration of an IL-6 receptor antibody after the initiation of cachexia essentially blocks the transition towards more severe body weight and muscle mass loss. Our direct examination of muscle protein turnover found that myofibrillar protein synthesis is repressed and ATP-dependent muscle protein degradation is increased during the initiation of cachexia. As circulating IL-6 levels surge and body weight loss intensifies, we report a further repression of myofibrillar protein synthesis that corresponds with repressed mTOR signaling that is not dependent on Akt regulation, but coincides with the activation of AMPK. Although we report a further induction of ATP dependent protein degradation during the progression of cachexia, the additional activation of ATP-independent protein degradation is also implicated as muscle mass loss becomes more severe. The activation of these protein degradation processes demonstrated IL-6 sensitivity, being repressed by the inhibition of the IL-6 receptor, which was able to block further muscle mass loss without any alleviation of repressed myofibrillar protein synthesis. Taken together, our data support the finding that the IL-6 receptor antibody treatment was effective in diminishing the rate of cachexia development, but not sufficient to reverse the process.

While the importance of activated muscle protein degradation during wasting has been well established, the relationship of muscle protein synthesis regulation to muscle mass loss during cachexia has demonstrated equivocal results [50], [51]. There is an overall trend in the literature to support that protein synthesis is reduced during the later stages of cachexia in both humans [8], [52], [53] and rodents [7], [54]. The first report measuring protein synthesis in mice bearing the MAC16 adenocarcinoma showed that synthesis was reduced during later stages of cachexia (>16% body weight loss), but not during initial stages [7]. We report myofibrillar protein synthesis is reduced during the initiation of cachexia, and is further repressed during the transition from initial to more severe loss. The inconsistency in findings related to our study at the initiation of cachexia may result from differences in the cachexia models. The MAC16 tumor causes rapid weight loss and progresses to severe cachexia in two weeks or less [7], [15]. In contrast, cachexia in the *Apc^Min/+^* mouse develops over a much longer time period [38]. Furthermore, the MAC16 tumor bearing mice in the study by Smith et al. [7] did not show a reduction in gastrocnemius muscle mass during the initial stages of body weight loss (0–10%), while we observed a reduction in gastrocnemius mass in *Apc^Min/+^* mice with minimal (≤5%) body weight loss. The reduction in myofibrillar protein synthesis did not appear related to decreased RNA abundance, since skeletal alpha actin mRNA expression was not changed throughout the progression of cachexia. In support of these data, we have also previously published muscle myosin heavy chain IIa and IIb mRNA expression was not affected by cachexia in the *Apc^Min/+^* mouse [40].

Although, the IGF-1/Akt/mTOR pathway is a well established regulator of muscle protein synthesis, it is well acknowledged that this pathway is a highly complex, tightly regulated cellular process with many potential sites of regulation [10]. The reduction in both circulating and muscle expression of IGF-1 has been shown in various wasting condition [11], [12], [13], [25], [55]. In the present study, we show that a reduction in muscle IGF-1 gene expression is an early event that occurs during the initiation of cachexia, and expression is further repressed as cachexia progresses. However, there is not a corresponding reduction in IGF-1 mRNA expression during the most severe period of wasting when muscle mass has reached a critically diminished state. While Akt activity is generally a marker of muscle growth, promoting protein synthesis through mTOR and inhibiting protein degradation through Foxo phosphorylation [56], we found the role of Akt signaling in the progression of muscle loss less certain. Despite the reduction in IGF-1 expression and mTOR signaling, we detected an increase in Akt phosphorylation as cachexia progressed. Previous studies have shown Akt activity during cachexia in tumor bearing rodents to be unchanged [25] or decreased [26]. Interestingly, we found the ability of Akt to phosphorylate its targets regulating muscle mass is compromised in cachectic muscle. Phosphorylation of Foxo was reduced during initial and intermediate stages of cachexia, and the phosphorylation of mTOR was repressed after the initiation of cachexia. Currently, there is limited explanation for this unconformity in Akt signaling activity. An explanation worth further examination could be related to Akt phosphorylation on Akt2, which is involved in glucose metabolism [57]. Or, in the *Apc^Min/+^* mouse, activated mTORC2 may phosphorylate Akt. Hyper phosphorylation of Akt has been reported in raptor KO mice [58]. Although we do not find a decrease in raptor expression with cachexia, we do observe that raptor phosphorylation is increased during the progression of cachexia. It is not certain if suppressed raptor function could result in hyperphosphorylation of Akt. The phosphorylation of mTOR target substrates, p70S6K and 4EBP1, corresponded with the reduction in myofibrillar protein synthesis throughout the progression of cachexia and are consistent with observations in other models of cancer cachexia [14], [15].

The energy sensitive AMPK pathway is an additional point of regulation in mTOR activity. AMPK is a negative regulator of cell growth, activated under circumstances of nutrient deprivation, energy depletion and cell stress [59]. AMPK activation can reduce muscle mTOR signaling and protein synthesis [60], [61], [62] through multiple mechanisms, including activation of TSC2 [23], [63], direct inhibition of mTOR itself [64], or inhibition of raptor [23]. Previous reports show tumor bearing rodents have a trend toward increased activation of muscle AMPK [25]. We report that AMPK activity, AMPK phosphorylation and raptor protein phosphorylation on the AMPK-specific Ser 792 site and increased during the transition from the initiation of cachexia to more intermediate body weight loss, and a further increased with extreme loss. These data are supported by previous studies showing AICAR-induced activation of AMPK resulted in a reduction in muscle protein synthesis associated with an increase in phosphorylation of raptor [62]. To our knowledge, alterations within the mTORC1 complex have not been examined in muscle during cachexia. Raptor has recently been shown to be an essential component of the mTORC1 complex. Raptor acts as a scaffolding protein to recruit downstream targets of mTOR, S6K and 4EBP1 [65], [66]. In skeletal muscle, raptor KO mice have a marked reduction in phosphorylation of both S6K and 4EBP1 [58]. The increase in muscle AMPK activity that corresponds with the acceleration of body weight loss could be due to surging IL-6 levels [67] and/or the reduction in muscle oxidative capacity found in severely cachectic *Apc^Min/+^* mice [40].

In both humans [68], [69], [70] and rodents cachexia [7], [31], [71], [72], [73] induces muscle proteolysis , and the ubiquitin dependent proteasomal system has been reported to be the dominant proteolytic pathway during cachexia [6]. Tumor bearing mice increase muscle protein degradation after 16% body weight loss [7]. Recent studies have supported these findings demonstrating an induction of the proteasomal system during later stages of cachexia, but not during initial stages of weight loss [69], [70], [74]. Gastric cancer patients showed an increase in ubiquitin gene expression with only 5% body weight loss [75]. We report an increase in ATP dependent protein degradation during the initial stages of weight loss and a further increase during intermediate and extreme weight loss. Discrepancies related to the contribution of muscle protein degradation during the initiation of cachexia are likely explained by the rate of development, the extent of weight loss, and the nature of underlying disease inducing the cachexia. The Foxo family of transcription factors have emerged as key regulators of muscle proteolysis through regulation of muscle specific E3 ligases atrogin1 and MuRF1 [10]. We have previously shown an increase in atrogin1 mRNA expression in *Apc^Min/+^* mice that had developed significant cachexia, while MuRF1 mRNA expression was not changed [39]. In the current study, the induction of atrogin and MuRF1 occurred differentially during the progression of cachexia. Atrogin1 expression was increased during the initiation of weight loss and further increased during the transition to more extensive loss, while MuRF1 did not increase until the most extreme body weight loss condition. *Apc^Min/+^* mice with extreme body weigh loss in our current study had significantly more pronounced weight loss and higher circulating IL-6 levels than that seen in our earlier study by Baltgalvis et al [39]. It has been proposed Foxo-induced transcription of atrogin1 and MuRF1 are differently regulated and MuRF1 transcription may involve additional co-factors [76]. In the current study, the increase in MuRF1 expression coincided with the largest increase in AMPK activity. AMPK has been previously shown to regulate expression of both atrogin1 and MuRF1 expression in muscle [77], [78].

While a role for autophagy/lysosomal degradation during muscle wasting has been widely acknowledged, the regulation and importance of this process during muscle wasting is still being explored. Rodent models of cachexia have shown increased expression of autophagy [26], [31] and lysosomal [31] related proteins. LPS-induced cachexia in mice increases a series of genes related to the autophagy process [27]. We provide further evidence for the increase in autophagy proteins during muscle wasting with cachexia. Furthermore, the induction of autophagy proteins appears to occur during the later stages of cachexia, as we found no expression of autophagy-related proteins during initial body weight loss. Additionally, autophagy related protein expression was blocked by IL-6r antibody administration. This is the first report, to our knowledge, demonstrating that muscle autophagy-related degradation is related to the progression of cachexia and circulating IL-6 levels. However, in cancer patients increased lysosomal enzyme activity was correlated with weight loss [79]. Previous reports using the IL-6r antibody have demonstrated a reduction in muscle protein breakdown by inhibition of proteasomal and lysosomal dependent pathways [37]. However, tumor bearing rats treated with suramin, a global cytokine inhibitor, demonstrated a suppression of muscle proteasomal activity [80]. It has also been shown that Foxo family proteins and the mammalian target of rapamycin (mTOR) can regulate the expression of autophagy genes [18]. We report a reduction in Foxo phosphorylation and trend for decreased mTOR phosphorylation during the initiation of cachexia that corresponded with an increase in proteasomal activation, but not autophagy-related expression, which suggests additional regulation of muscle autophagy gene expression beyond Foxo3 and mTOR. IL-6r antibody treatment in *Apc^Min/+^* mice attenuates muscle proteolysis by reducing the expression of both proteasomal and autophagy-related proteins.

In summary, our data show muscle protein synthesis and related IGF-1 signaling is suppressed during the initial progression of cachexia in the *Apc^Min/+^* mouse. The decrease in IGF-1 signaling is accompanied by a paradoxical increase in Akt phosphorylation, despite a reduction in downstream mTOR and FOXO signaling. In contrast to IGF-1 expression, AMPK activation was increased throughout later stages of cachexia, which might explain the decrease in mTOR signaling during extreme body weight loss. In regards to protein degradation, we show protein degradation was due, in part, by the increased ubiquitin proteasomal activation and lysosomal/autophagy pathways. However, the regulation of the two systems appears to be different as cachexia severity does not activate both systems equally. Protein degradation during the initial stages of cachexia appears to be the result of increased ubiquitin proteasomal activation independent of autophagy/lysosomal activation. However, later stages of cachexia increase protein degradation through proteasomal and autophagy/lysosomal pathways. Lastly, inhibition of IL-6 signaling in *Apc^Min/+^* mice with initial body weight loss can prevent the progression into late stage cachexia independently of changes in protein synthesis. This is associated with the attenuation of proteasomal activation and prevention of autophagy-related protein expression, maintaining what is observed during the initial stages of cachexia.

## Supporting Information

Figure S1 **Muscle wasting in cachectic** ***Apc^Min/**+**^*** **mice is associated with a reduction in muscle mass, protein synthesis and IGF-1 expression.** Wild-type and *Apc^Min/+^* mice were sacrificed at 12 and 20 weeks of age. A) Gastrocnemius muscle mass. B) Myofibrillar protein synthesis. C) IGF-1 mRNA expression normalized to 12 week wild-type mice. Values are means ± SE. Significance was set at p<0.05. * Signifies different from 12 week mice within genotype. Gastroc, Gastrocnemius.
(TIF)

Figure S2 **AMPK signaling is increased in cachectic** ***Apc^Min/**+**^*** **mice.** A). *Upper* representative western blot of phosphorylated and total forms of AMPK. *Lower* The ratio of phosphorylated and total AMPK expression normalized to 12 week wild-type mice. B). Muscle AMPK activity normalized to 12 week wild-type mice. C). *Upper* representative western blot of phosphorylated and total forms of raptor. *Lower* The ratio of phosphorylated and total raptor expression normalized to 12 week wild-type mice. Values are means ± SE. Significance was set at p<0.05. *Signifies difference within genotype.
(TIF)

Figure S3 **Rates of ATP-independent and ATP-dependent protein degradation are similar between wild-type and weight stable** ***Apc^Min/**+**^*** **mice.** Protein degradation measurements were taken at 12 weeks of age for both wild-type and *Apc^Min/+^* mice.
(TIF)

Figure S4 **Autophagy is increased in cachectic** ***Apc^Min/**+**^*** **mice.** A). *Upper* representative western blot of Beclin-1 protein. *Lower* Quantification of Beclin-1 protein normalized to 12 week wild-type mice, B). *Upper* representative western blot of Atg7 protein. *Lower* Quantification of Atg7 protein. Values are means ± SE. Significance was set at p<0.05. * Signifies difference from 12 week mice within genotype.
(TIF)

Figure S5 **Administration of IL-6 receptor antibody did not affect food intake in wild-type or** ***Apc^Min/**+**^*** **mice.**
(TIF)

Figure S6 **Administration of IL-6 receptor antibody did not affect total polyp number in** ***Apc^Min/**+**^*** **mice. Polyp counts were taken at 18 weeks of age.**
(TIF)
