# Supplementary figures and images for: The Regulation of Skeletal Muscle Protein Turnover during the Progression of Cancer Cachexia in the ApcMin/+ Mouse
Source: PLoS One. 2011 Sep 19;6(9):e24650. doi: 10.1371/journal.pone.0024650 (PMC3176277; doi:10.1371/journal.pone.0024650)

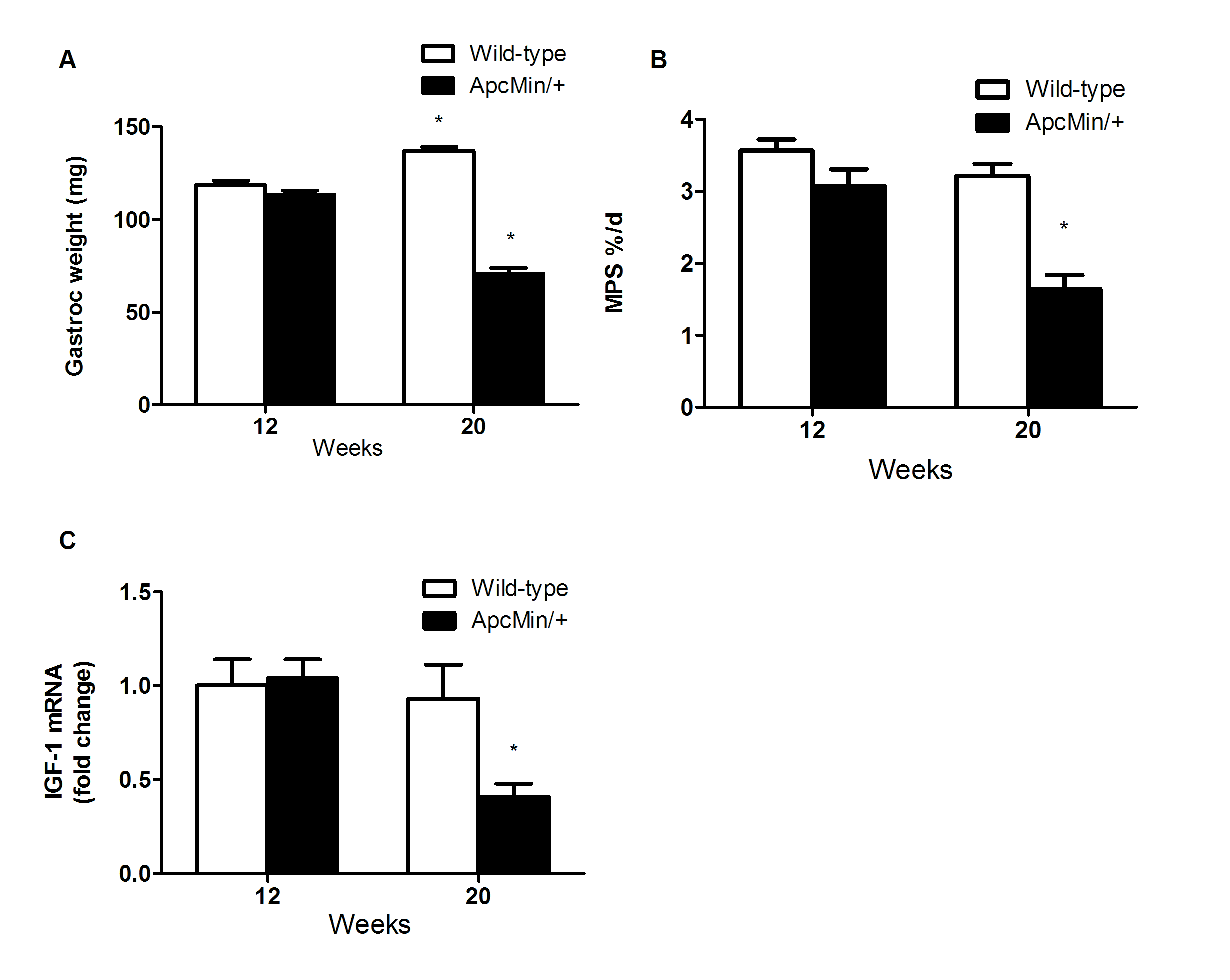

Supplement: Figure S1 — Muscle wasting in cachectic ApcMin/+ mice is associated with a reduction in muscle mass, protein synthesis and IGF-1 expression. Wild-type and ApcMin/+ mice were sacrificed at 12 and 20 weeks of age. A) Gastrocnemius muscle mass. B) Myofibrillar protein synthesis. C) IGF-1 mRNA expression normalized to 12 week wild-type mice. Values are means ± SE. Significance was set at p<0.05. * Signifies different from 12 week mice within genotype. Gastroc, Gastrocnemius. (TIF) [file pone.0024650.s001.tif]

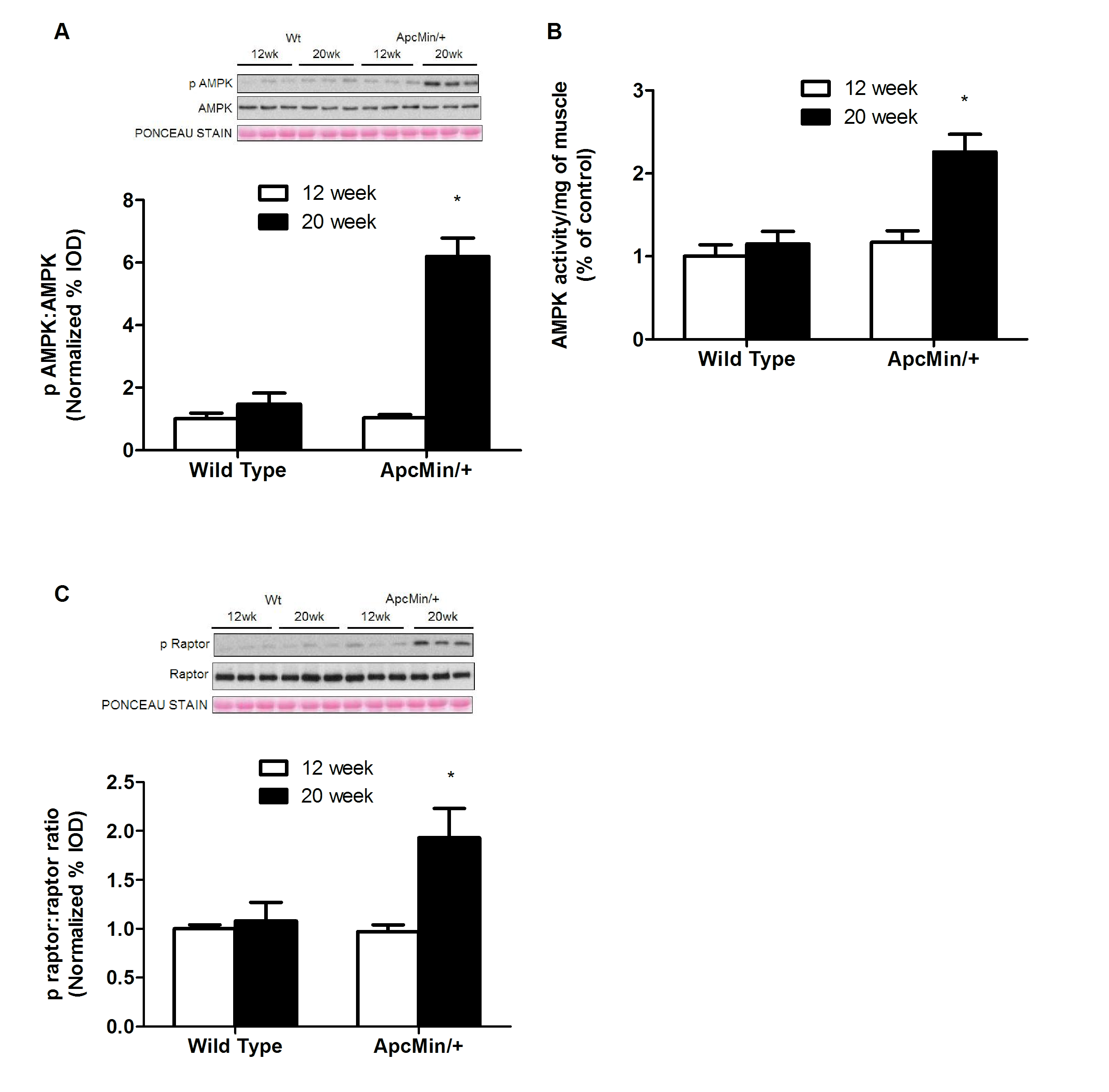

Supplement: Figure S2 — AMPK signaling is increased in cachectic ApcMin/+ mice. A). Upper representative western blot of phosphorylated and total forms of AMPK. Lower The ratio of phosphorylated and total AMPK expression normalized to 12 week wild-type mice. B). Muscle AMPK activity normalized to 12 week wild-type mice. C). Upper representative western blot of phosphorylated and total forms of raptor. Lower The ratio of phosphorylated and total raptor expression normalized to 12 week wild-type mice. Values are means ± SE. Significance was set at p<0.05. *Signifies difference within genotype. (TIF) [file pone.0024650.s002.tif]

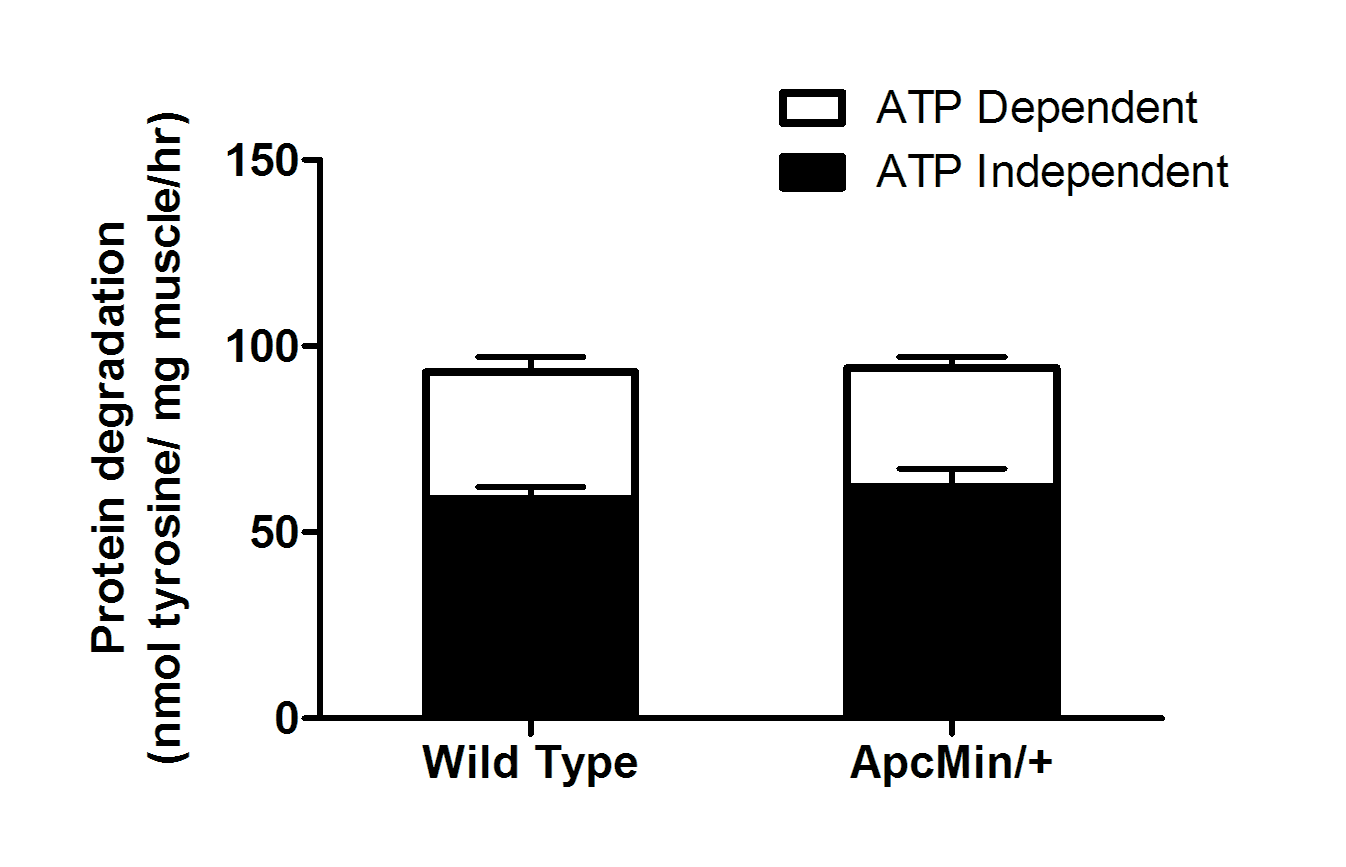

Supplement: Figure S3 — Rates of ATP-independent and ATP-dependent protein degradation are similar between wild-type and weight stable ApcMin/+ mice. Protein degradation measurements were taken at 12 weeks of age for both wild-type and ApcMin/+ mice. (TIF) [file pone.0024650.s003.tif]

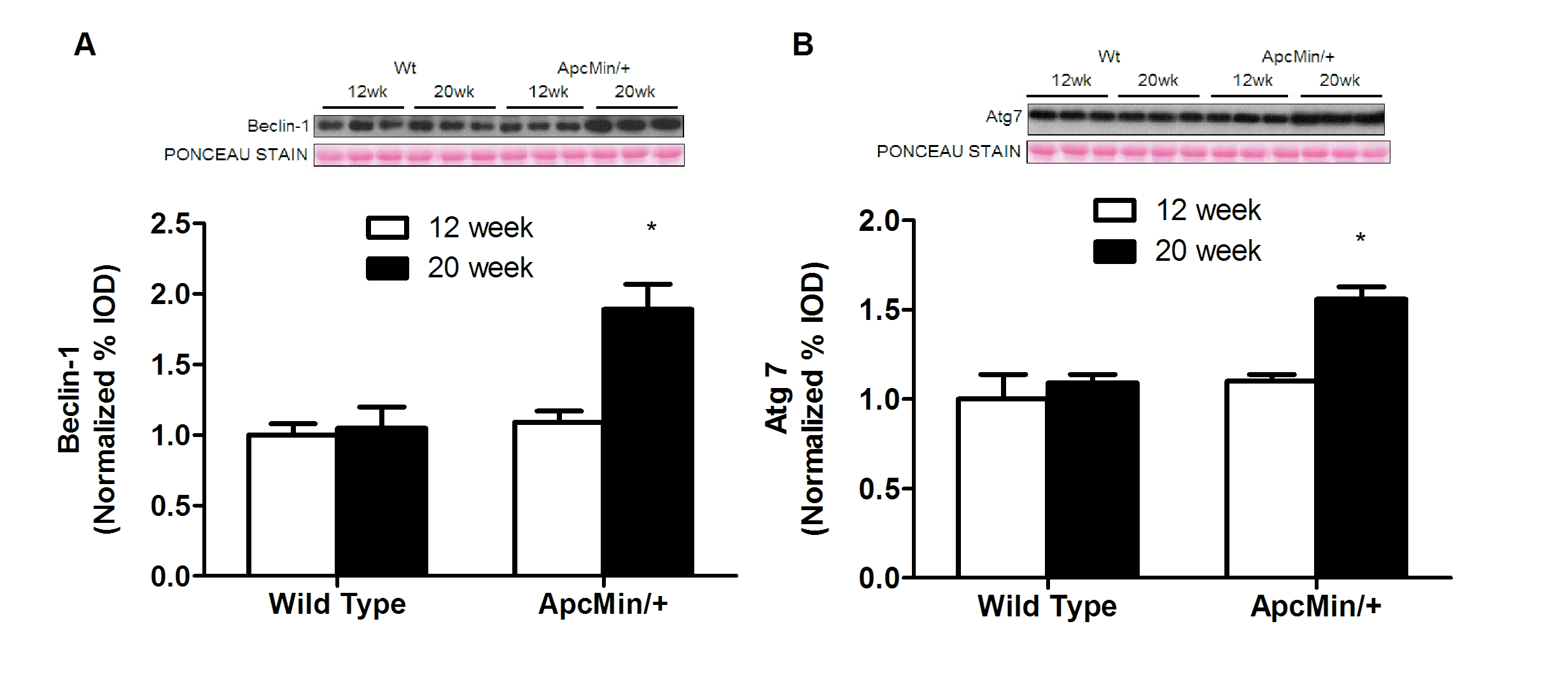

Supplement: Figure S4 — Autophagy is increased in cachectic ApcMin/+ mice. A). Upper representative western blot of Beclin-1 protein. Lower Quantification of Beclin-1 protein normalized to 12 week wild-type mice, B). Upper representative western blot of Atg7 protein. Lower Quantification of Atg7 protein. Values are means ± SE. Significance was set at p<0.05. * Signifies difference from 12 week mice within genotype. (TIF) [file pone.0024650.s004.tif]

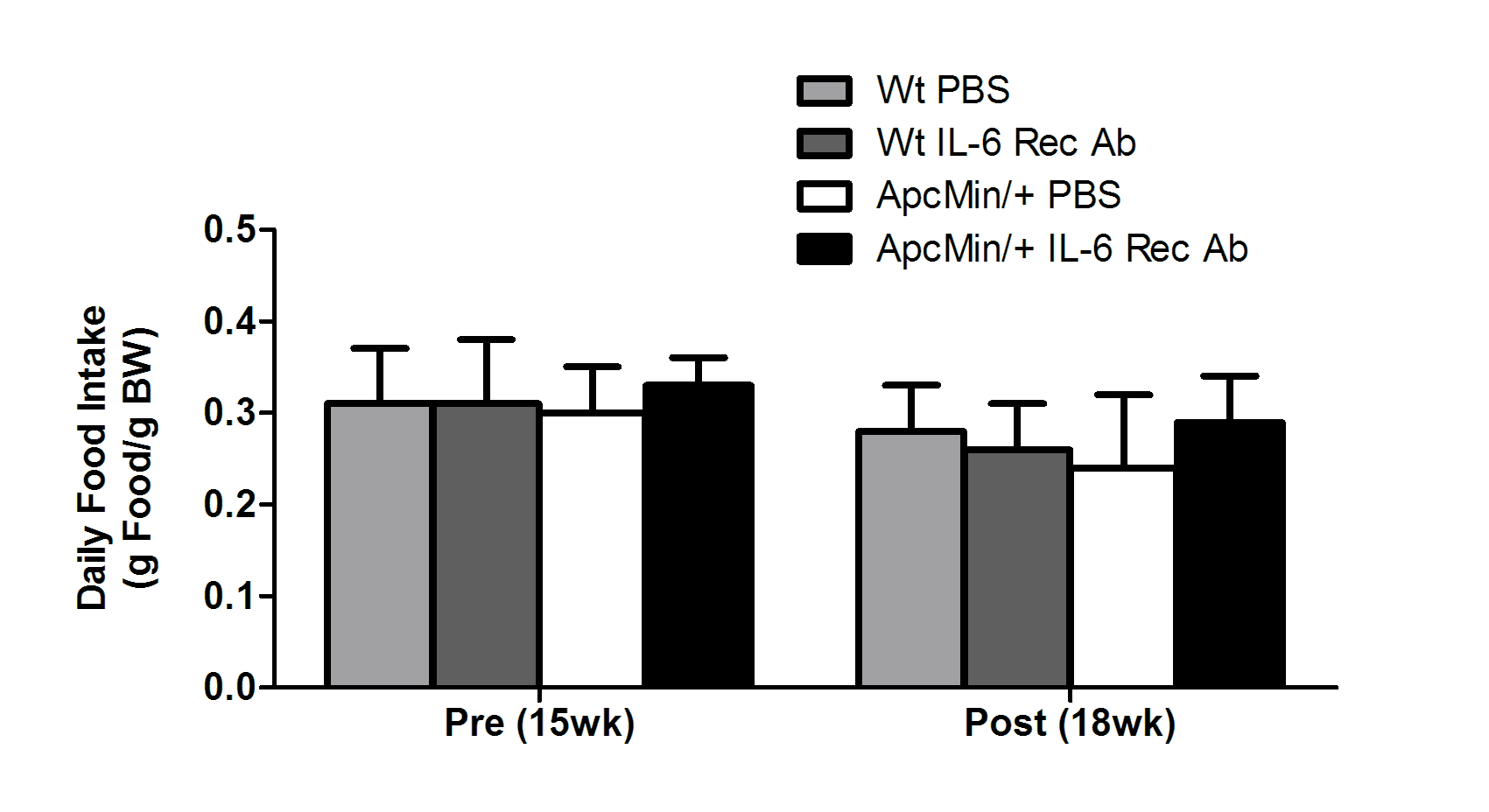

Supplement: Figure S5 — Administration of IL-6 receptor antibody did not affect food intake in wild-type or ApcMin/+ mice. (TIF) [file pone.0024650.s005.tif]

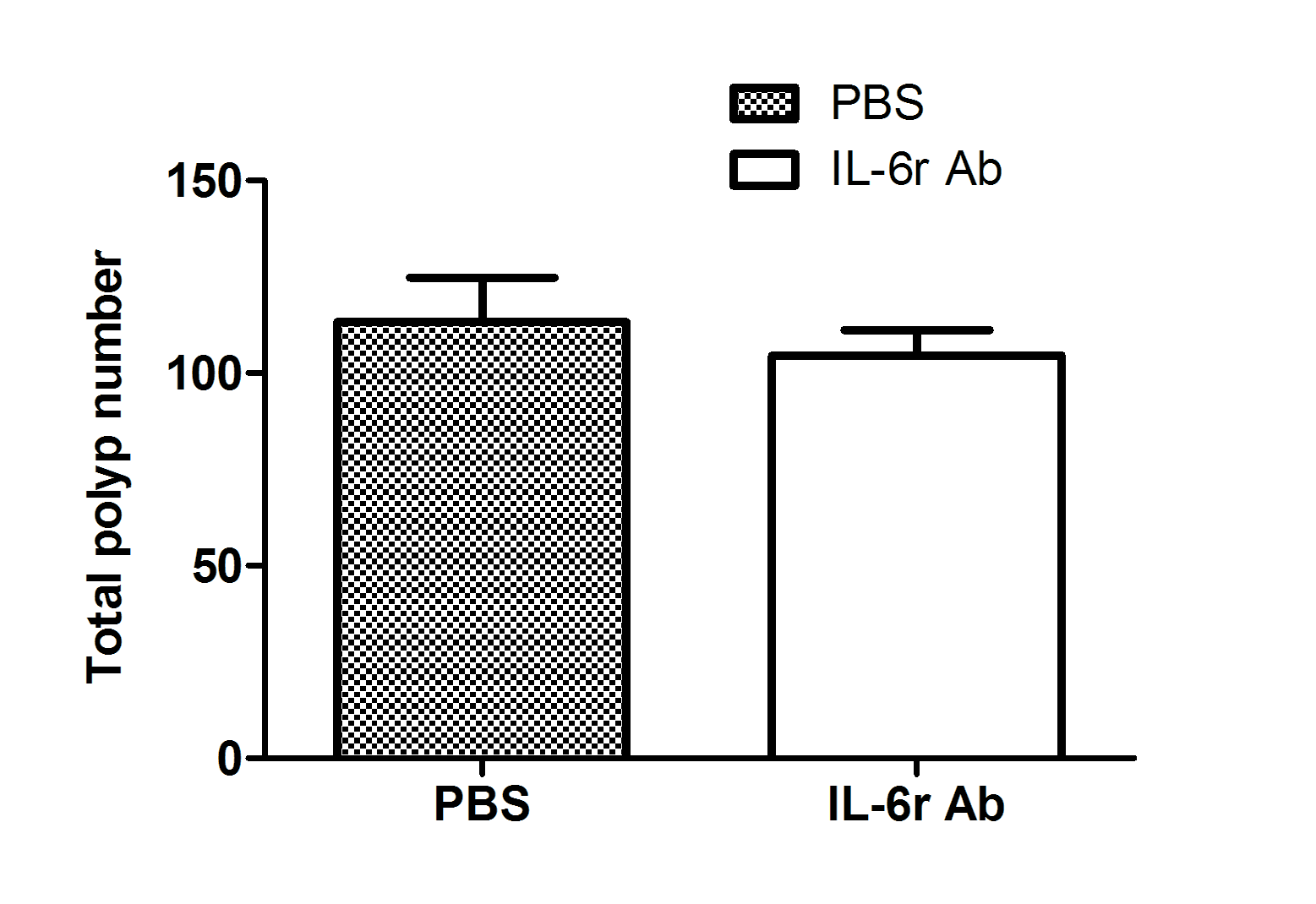

Supplement: Figure S6 — Administration of IL-6 receptor antibody did not affect total polyp number in ApcMin/+ mice. Polyp counts were taken at 18 weeks of age. (TIF) [file pone.0024650.s006.tif]
